# Effect of β-hydroxy-β-methylbutyrate (HMB) on the Muscle Strength in the Elderly Population: A Meta-Analysis

**DOI:** 10.3389/fnut.2022.914866

**Published:** 2022-07-13

**Authors:** Ziru Lin, Anqi Zhao, Jiguo He

**Affiliations:** China Agricultural University, Beijing, China

**Keywords:** sarcopenia, HMB, muscle strength, meta-analysis, elderly population

## Abstract

**Objective:**

This meta-analysis aims to evaluate the effect of the emerging nutritional ingredient β-hydroxy-β-methylbutyrate (HMB) on the muscle strength of elderly people.

**Methods:**

Computer systems-based search and sorting of relevant documents published before December 2020 in the China Journal Full-text Database (CNKI), Wan Fang Database (Wan Fang), VIP Chinese Science and Technology Journal Database (VIP), PubMed, Web of Science, and EMbase Database was done. Two researchers independently screened the literature based on the inclusion and exclusion criteria and performed data extraction and quality evaluation. Rev Man 5.X software was used for systematic review and meta-analysis.

**Results:**

A total of 9 randomized controlled trials (RCT) studies were included in the study, which comprised 896 subjects. The overall impact on muscle strength-related indicators (SMD = 0.41; 95% CI: 0.28, 0.54); *p* < 0.00001) was statistically significant. Conclusion: Supplementation of HMB and preparations containing HMB ingredients aid in increasing muscle strength in the elderly population.

Sarcopenia is a progressive loss of skeletal muscle mass associated with aging leading to decreased muscle strength and/or muscle function (1). This can negatively impact the health of elders, resulting in an increased risk of chronic metabolic diseases, physical disability, falls and fractures, and decreased self-dependence (1). β-hydroxy β-methylbutyric, abbreviated as HMB, is a product of leucine metabolism in the body (2). The Ministry of Health of China listed it in the 2011 announcement no. 01 Into the new resource food catalog (3). Due to its excellent properties of promoting muscle protein synthesis and inhibiting muscle protein breakdown, HMB has long been used as a nutritional supplement for improving athletes' muscle mass and performance. With increasing research in recent years, HMB has also been found to improve muscle wasting and promote wound healing, which is gaining attention in the medical field (2). Some Western studies have shown that the prevalence of sarcopenia is 20% in the elderly aged 65 and over and 50% ~ 60% in the population aged 80 and over (4). Therefore, the European Working Group on sarcopenia in the older people (ewgsop) recommends that the elderly aged 65 and over should be routinely checked for sarcopenia. Sarcopenia can cause falls, fractures, movement disorders, etc., which increase the risk of disability and loss of self-care ability, in the vulnerable popluation. It has been estimated that in 2,000, the direct medical expenses spent on sarcopenia patients in the United States were close to $1.85 billion, accounting for 1.5% of the total medical expenses (5). Sarcopenia severely affects the physical and mental health of the elderly and is an important disease causing a decline in quality of life. Actively carrying out prevention and treatment has important social significance and economic benefits. Irrespective of whether the subjects were in-hospitalized elderly or healthy elderly people, earlier studies have shown that HMB supplementation is beneficial to improving muscle quality and muscle function, while there was no change in the control group (6–9).

Therefore, This article aims to explore the effect of HMB on the muscle strength of elderly people using the method of meta-analysis.

## Materials and Methods

### Literature Inclusion Criteria

(1) Any randomized controlled trials using HMB or HMB-containing preparations as intervention were considered for this meta-analysis.

(2) The summary results of the study are complete and reflected truthfully, and the outcome indicators can be expressed by corresponding statistical indicators.

(3) The studies should provide clear information about the research type, research method, number of cases, and number of cases lost to follow-up.

### Literature Source

The literature search was conducted in the Chinese Journal Full-text Database (CNKI), Wan Fang Database (Wan Fang), VIP Chinese Science and Technology Journal Database (VIP), PubMed, Web of Science, EMbase Database to identify eligible studies published through December 2020. And perform manual search and completion of references that meet the criteria in the reading literature and supplement relevant literature as much as possible.

### Search Strategy

CNKI search strategy:

SU =(sarcopenia + amyotrophic)^*^ (β-hydroxy β-methyl butyrate + hydroxy methyl butyrate +b-hydroxy-b-methyl butyrate + beta-hydroxy-beta-methyl butyrate + HMB)

PubMed search strategy:

#1“sarcopenia” [Title/Abstract] OR “sarcopenias” [Title/Abstract] OR“Muscle wasting and aging” [Title/Abstract] OR “Muscle-wasting” [Title/Abstract] OR “muscle loss” [Title/Abstract]

#2“HMB” [Title/Abstract] OR “β-hydroxy-β-methylbutyrate”[Title/Abstract] OR“beta-hydroxyisovaleric acid” [Title/Abstract] OR “beta-hydroxy beta-methylbutyrate” [Title/Abstract] OR “HMB-d6” [Title/Abstract] OR “HMB-Ca” [Title/Abstract] OR “b-hydroxy-b-methylbutyrate” [Title/Abstract]

#3 #1 AND #2

### Literature Screening and Data Extraction

**Figure 1:**
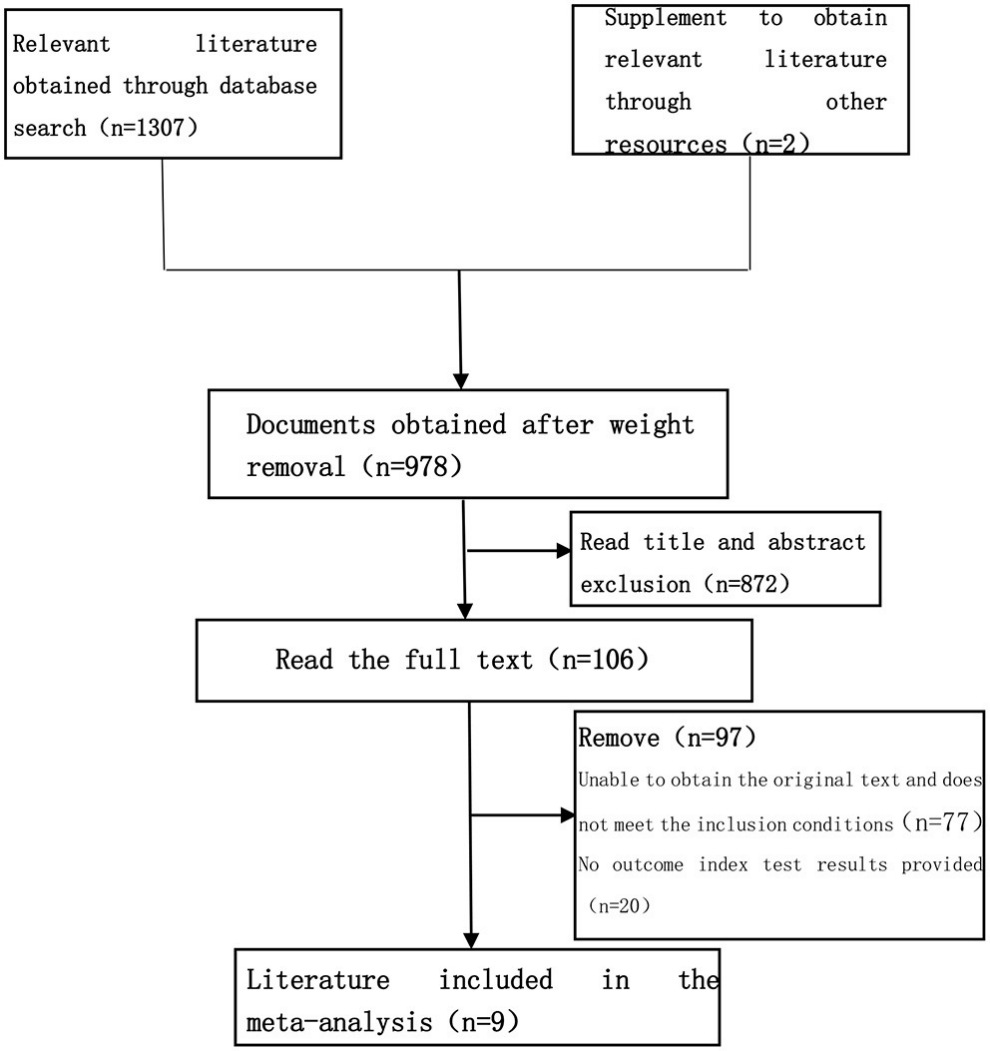
Document screening flow chart.

The studies were sorted out by the computer, and the duplicate entries were removed. Studies not fulfilling the inclusion criteria and those that meet the exclusion criteria are excluded. The study records obtained from the computer searches were imported to EndNote X9. Two reviewers were independently responsible for screening the documents, extracting the data, cross-checking, and screening out non-conformances. In case of any discrepancies between the reviewers, disagreements were resolved by consensus or consultation with the senior reviewer (third party). The file filtering process is shown in Figure 1.

From the selected studies following data were extracted:

(1) General study characteristics: Author, type of study, year of publication, number of subjects, age, gender, nationality, intervention plan, and placebo;
(2) Outcome indicators: formulated a data extraction table, extracted the sample size of the experimental group and the control group, the results of the indicators were involved in the research, and the content of the above outcome indicators;
(3) Classify and convert the indicators included in the study to obtain usable data.

### Quality Evaluation

The RevMan 5.3 software was used for quality evaluation. According to the evaluation content of the risk of bias assessment tool (Recommended by Cochrane Handbook 5.1), an independent evaluation of the quality of the included analysis was carried out. The evaluation content is shown in Table 1.

**Table 1:** The main content of quality evaluation.

**Rating entries**	**Evaluation content**
Random sequence generation	Is the random sequence generation method correct?
Assign hidden	Is the distribution of interventions well hidden?
Investigator-subject blinded	Whether the grouping was known in advance to the investigator and subjects?
Blinded evaluation of study outcomes	Does it describe the implementation of blinding of evaluators?
Closing Data Integrity	Data completeness, whether the number of missed visits and withdrawals, their causes, and disposition are reported?
Selective reporting of study results	Does it include all the pre-defined results?
Other biases	Other possible risks

#### Heterogeneity Test

In this study, the Q statistic test was performed to assess the heterogeneity between the results of the included studies. If *p* > 0.1, then there is no significant presence of heterogeneity (between the results of the included studies), and the analysis tool should adopt a fixed-effects model; When *p* ≤ 0.10, then it is considered as there is a presence of heterogeneity. The percentage change between studies indicating heterogeneity was reported using I^2^ values, and the interpretation is as follows:

Severe heterogeneity: 75% ≤ I^2^ < 100%;
Moderate heterogeneity: 50% ≤ I^2^ < 75%;
Mild heterogeneity: 25% ≤ I^2^ < 50%;
No heterogeneity: 0% ≤ I^2^ < 25%.

In the case of heterogeneity, the reasons for heterogeneity should be identified, and the accuracy of the original data and data extraction technology should be checked. If necessary, a subgroup analysis of each study can be carried out to eliminate confounding factors and reduce the heterogeneity within the research. The cause of heterogeneity can be judged by sensitivity analysis, the presence or absence of changes in heterogeneity, combined analysis results, or the state of change can be observed, and the stability (Meta results) evaluation can be completed on this basis.

#### Publication Bias

Compared to the negative and non-statistically significant research results, statistically significant results are more likely to be published or reported, resulting in publication bias. Publication bias can be qualitatively observed by visual inspection of the funnel chart.

### Statistical Analysis

The Meta-analysis part uses RevMan 5.3 software recommended by Cochran Library. Effect size measurements are expressed as standardized mean difference (SMD) with a confidence interval of 95%.

The result criterion is: (1) If the 95% CI of the effect size contains zero or is directly zero, and the diamond box in the forest diagram (representing the combined effect size) crosses the invalid line, then there is no statistical difference in the effect of intervention measures on the relevant outcome indicators; (2) When the effect size is >0 or the lower limit of 95% CI is >0, and the diamond box is positioned to the right side of the equivalence line, then the level of the intervention measures on the relevant outcome indicators is set to *p* < 0.05; (3) If the upper limit of effect size (95% CI) or itself is smaller than zero, and the diamond box is positioned to the left side of the equivalence line, then the level of the intervention measures on the relevant outcome indicators (the level of the meta-analysis test) is set to *p* = 0.05.

## Results

### Literature Search Results

Using a pre-established search strategy, a computer system was used for the literature search regarding the use of HMB as an intervention of sarcopenia within the well-known domestic and foreign journal websites. A total of 1,307 related documents were searched. Finally, a total of 9 articles on muscle strength were included in this study. The RCT trial literature on related intervention indicators was all in English, with clear outcome indicators and trial results, which met the conditions for meta-analysis. The basic characteristics of the included studies are shown in Table 2.

**Table 2:** Characteristics of include studies.

**Research**	**Country**	**Sample volume**	**State of health**	**Age**	**Sex**	**Intervention group supplement**	**Control supplement**	**Physical exercise**	**Intervention duration**
**Nasimi** **2020**	Iran	I = 33 C = 31	Sarcopenia patients(AWGS)	65+	I:M = 27 C:M = 23	300 g yogurt(3 g HMB;1000IU Vitamin D,500 mg Vitamin C)	300 g yogurt	Nix	12 weeks
**Riera** **2020**	South America	I = 33 C = 33	Mild sarcopenia or/and patients with knee osteoarthritis	63.5 ±9.6	FM = 85.5%	Creatine, glutamine, HMB	Analgesics	Exercise in the hospital	12 weeks
**Malafarina** **2017**	Spain	I = 36 C = 38	Elderly hip fracture	65+	I:M16,F33 C:M8,F35	HMB 3 g, 36 g protein,2000 IU Vitamin D	Routine nutrition	Nix	During the hospital
**Ekinci** **2016**	Turkey	I = 32 C = 30	Elderly women with hip fractures	65+	Females	HMB 3 g,36 g protein,2000 IU Vitamin D	Routine nutrition	Nix	30 days
**Nishizaki** **2015**	Japan	I = 12 C = 11	Elderly after knee surgery	70.5	Not mentioned	2 × (HMB 1.5 g, L-Arginine 7 g, L-glutamine 7 g)	Conventional treatment	Nix	14 days
**Fitschen** **2016**	America	I = 16 C = 17	Hemodialysis patients	I:57 ±8 C:53 ±13	Not mentioned	3 × HMB 1 g	Placebo	Nix	24 weeks
**Berton** **2015**	Italy	I = 32 C = 33	Healthy elderly women	69.5 ±5.3	Females	1.5 g/d Ca-HMB	Regular diet	Exercise in the hospital twice a week	8 weeks
**Olveira** **2015**	Spain	I = 15 C = 15	Patients with bronchiectasis	56.1 ±13	FM = 18	1.5 g HMB+18 g protein	Standard diet	Exercise in the hospital twice a week	12 weeks
**Stout** **2013**	America	I = 22 C = 21	Healthy elderly over 65	65+	I:M12,F13 C:M14,F11	3.0 g/d Ca-HMB 8 g/d carbohydrate	Placebo	Nix	24 weeks

### Quality Evaluation of Included Studies

**Figure 2:**
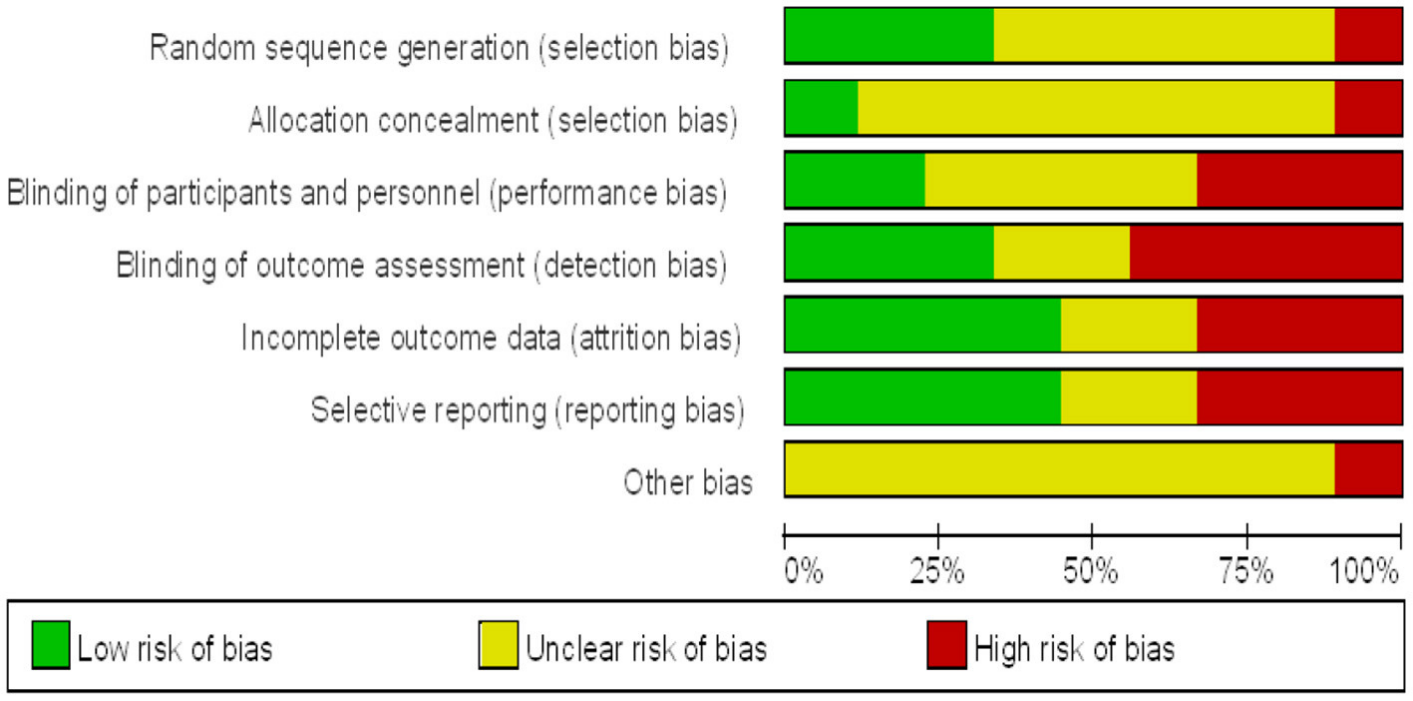
Proportion of risk of bias included in muscle strength studies.

**Figure 3:**
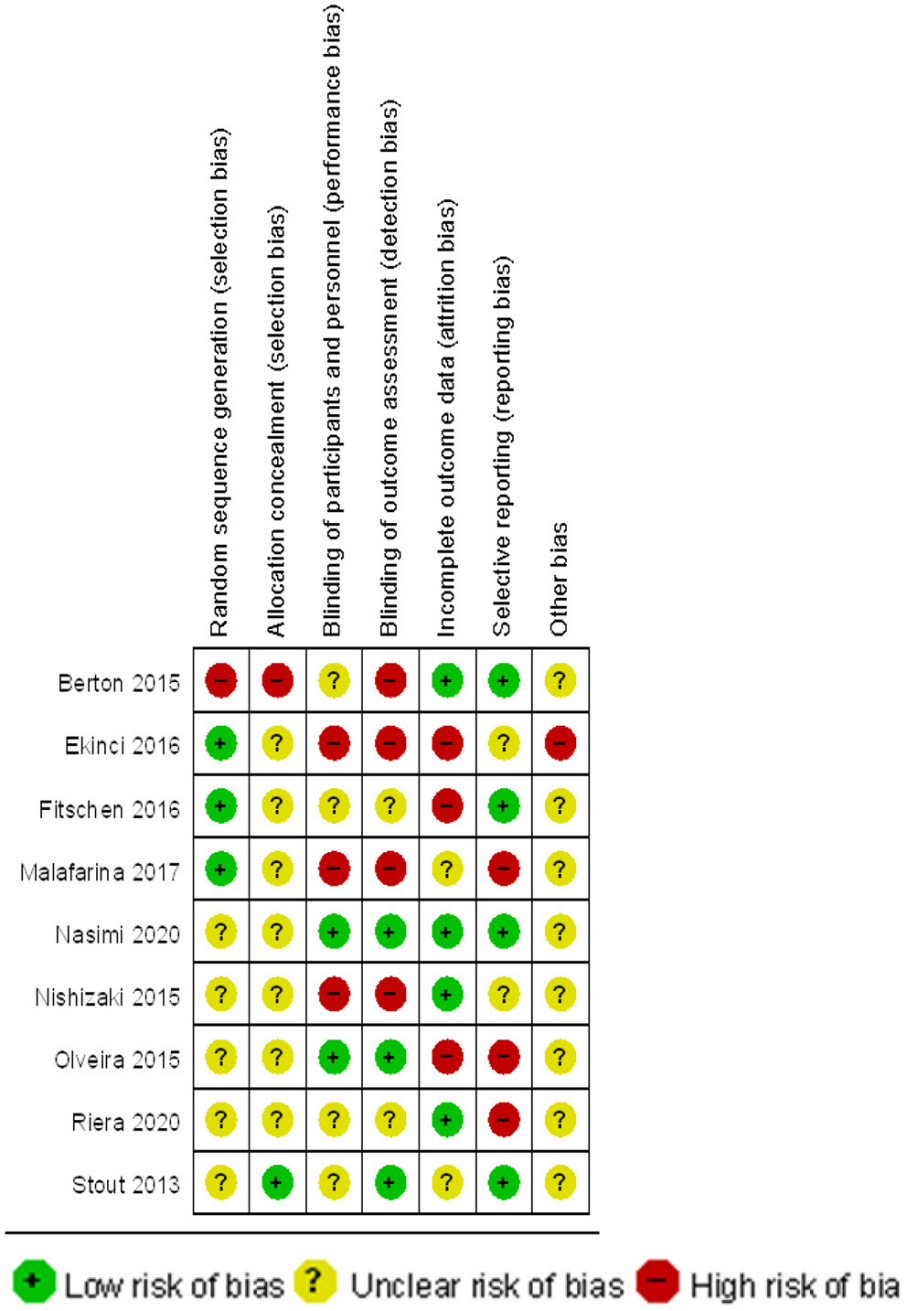
Summary chart of risk of bias for inclusion on muscle strength studies.

The quality of the included studies was evaluated separately (Figures 2, 3). The results show that the studies selected based on the inclusion and exclusion criteria are of high quality and can be used for meta-analysis.

### Publication Bias

**Figure 4:**
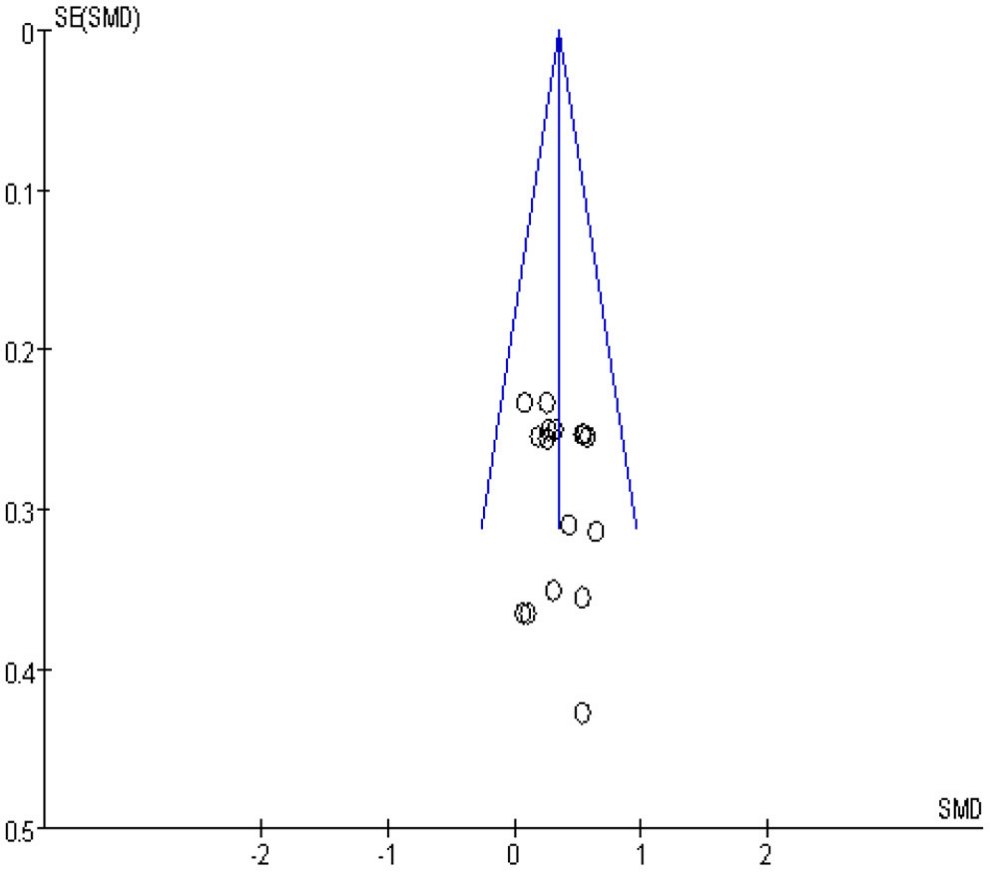
Muscle force publication bias funnel plot.

The analysis for the risk of publication bias was performed for the primary outcome indicator muscle strength, a funnel plot was plotted as in Figure 4, and it was observed that the likelihood of a case of publication bias was low in the studies on muscle strength.

### Upper Limb Muscle Strength

**Figure 5:**
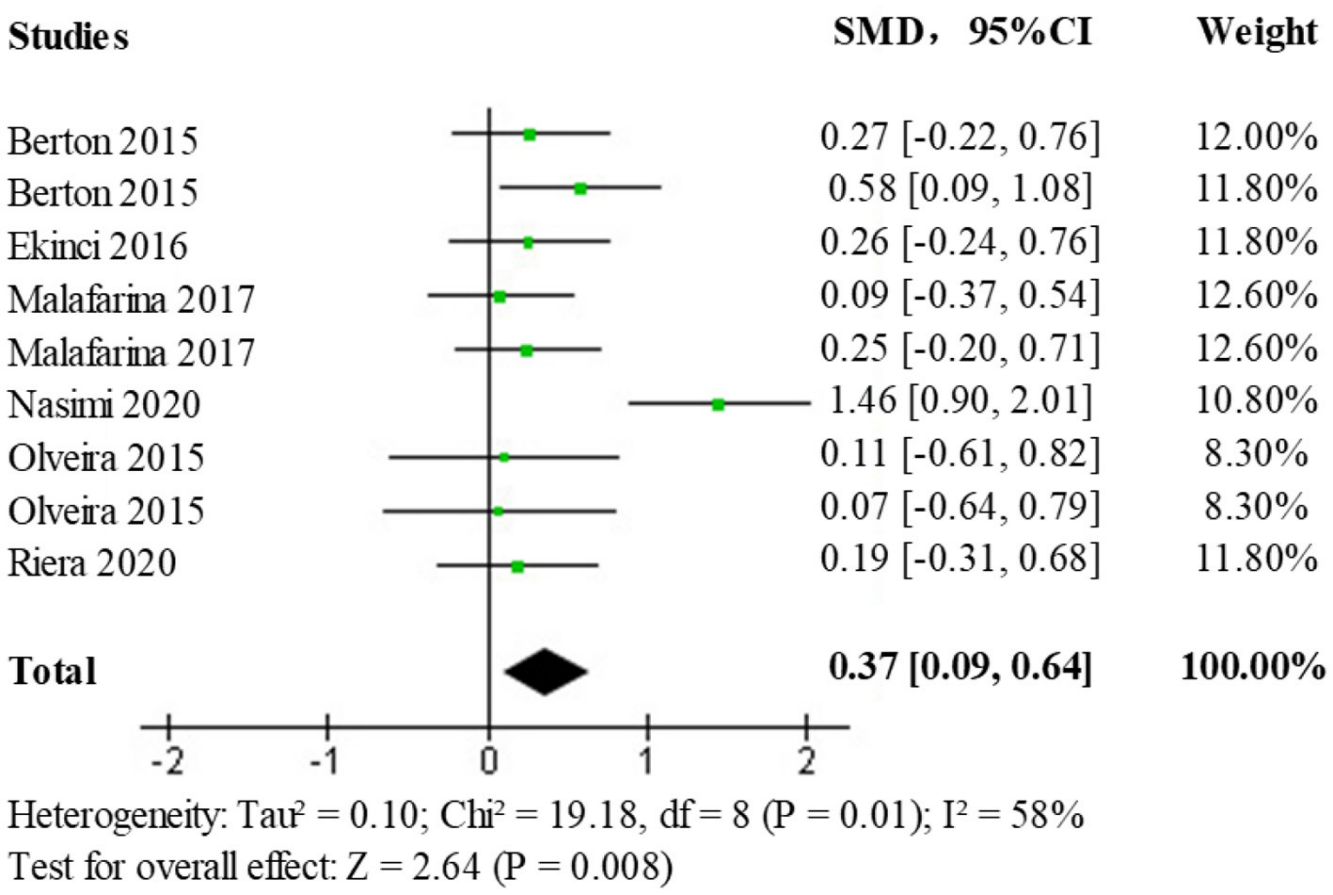
Forest plot of the effect of HMB on upper limb muscle strength in the elderly population.

In the selected literature, upper extremity muscle strength data have been obtained primarily by testing grip strength, grip duration, and isometric/isometric flexion and extension of the elbow joint. A heterogeneity test (*p* = 0.01, I^2^ = 58%) comparing the upper limb muscle strength changes between the HMB intervention and control groups found a moderate statistical heterogeneity between studies, and a further heterogeneity test is needed to clarify the main sources of heterogeneity as well as the stability and reliability of these findings. Results obtained using a random-effects model (SMD = 0.37; 95% CI: 0.09, 0.64; *p* < 0.008) showed statistically significant differences (*p* < 0.050) among the treatment populations, the forest plot is shown in Figure 5, indicating a facilitative effect of HMB on upper limb muscle strength.

**Figure 6:**
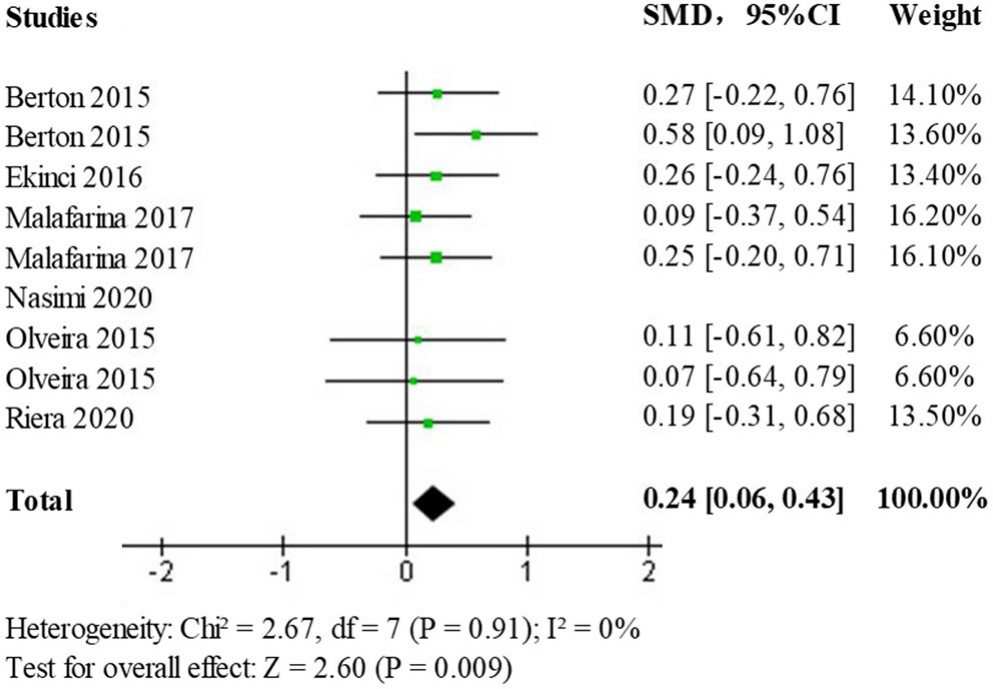
Forest plot of the effect of HMB on upper limb muscle strength after heterogeneous treatment in the elderly population.

Due to the presence of moderate heterogeneity, a heterogeneity test was performed to remove the results of each study one by one using observation funnel plots, by removal of single studies, and after the reanalysis operation, it was found that the study by Nasimi et al. was the main source of heterogeneity. After removing the data from this study and reanalyzing it, the heterogeneity test (*p* = 0.91, I^2^ = 0%) showed no statistically significant heterogeneity. The effect size results (SMD = 0.24; 95% CI: 0.06, 0.43; *p* < 0.009) after heterogeneous treatment in the elderly population shows that the new analysis did not change significantly from the original analysis indicating stable and reliable results, the forest plot is shown in Figure 6.

### Lower Extremity Muscle Strength

**Figure 7:**
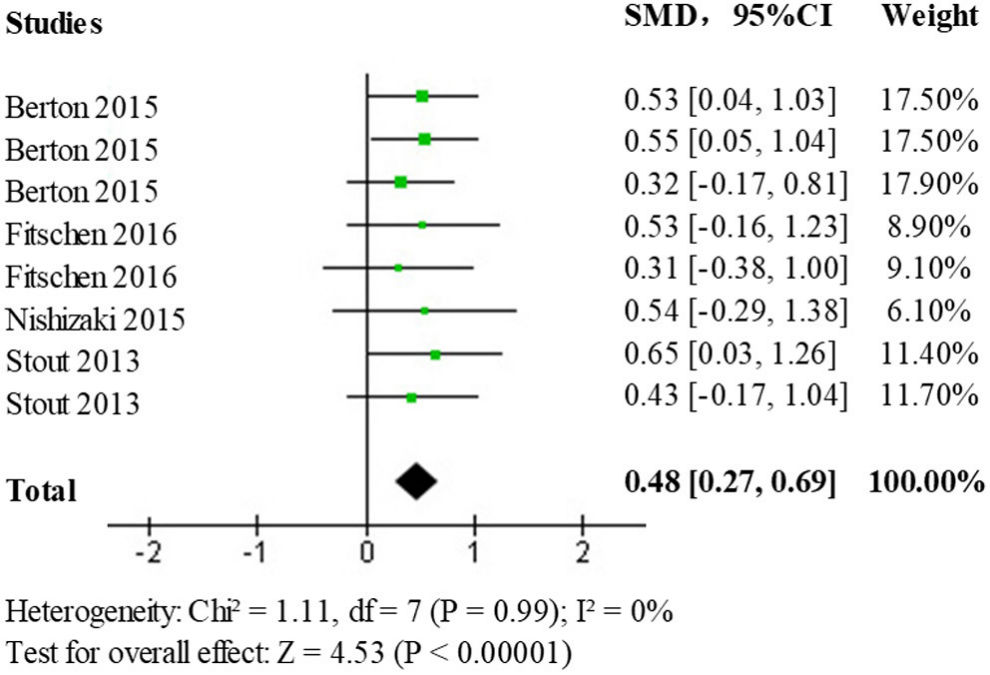
Forest plot of the effect of HMB on lower limb muscle strength in the elderly population.

In the selected literature, Lower extremity muscle strength data was obtained from 60° isometric/isometric knee flexion, and extension, 180° isometric/isometric knee flexion and extension, and chair stand testing. Data on the effects of the HMB intervention compared to controls on lower extremity muscle strength were analyzed using standardized mean difference (SMD) as the main effect measure. A test of heterogeneity (*p* = 0.99, I^2^ = 0%) found no statistically significant heterogeneity between studies. Results obtained using a fixed-effects model (SMD = 0.48; 95% CI: 0.27, 0.69; *p* < 0.00001) showed statistically significant differences (*p* < 0.05) among the treatment population, the forest plot is shown in Figure 7. This indicates that HMB has a promotional effect on lower limb strength.

### Analysis of Overall Muscle Strength Indicators

**Figure 8:**
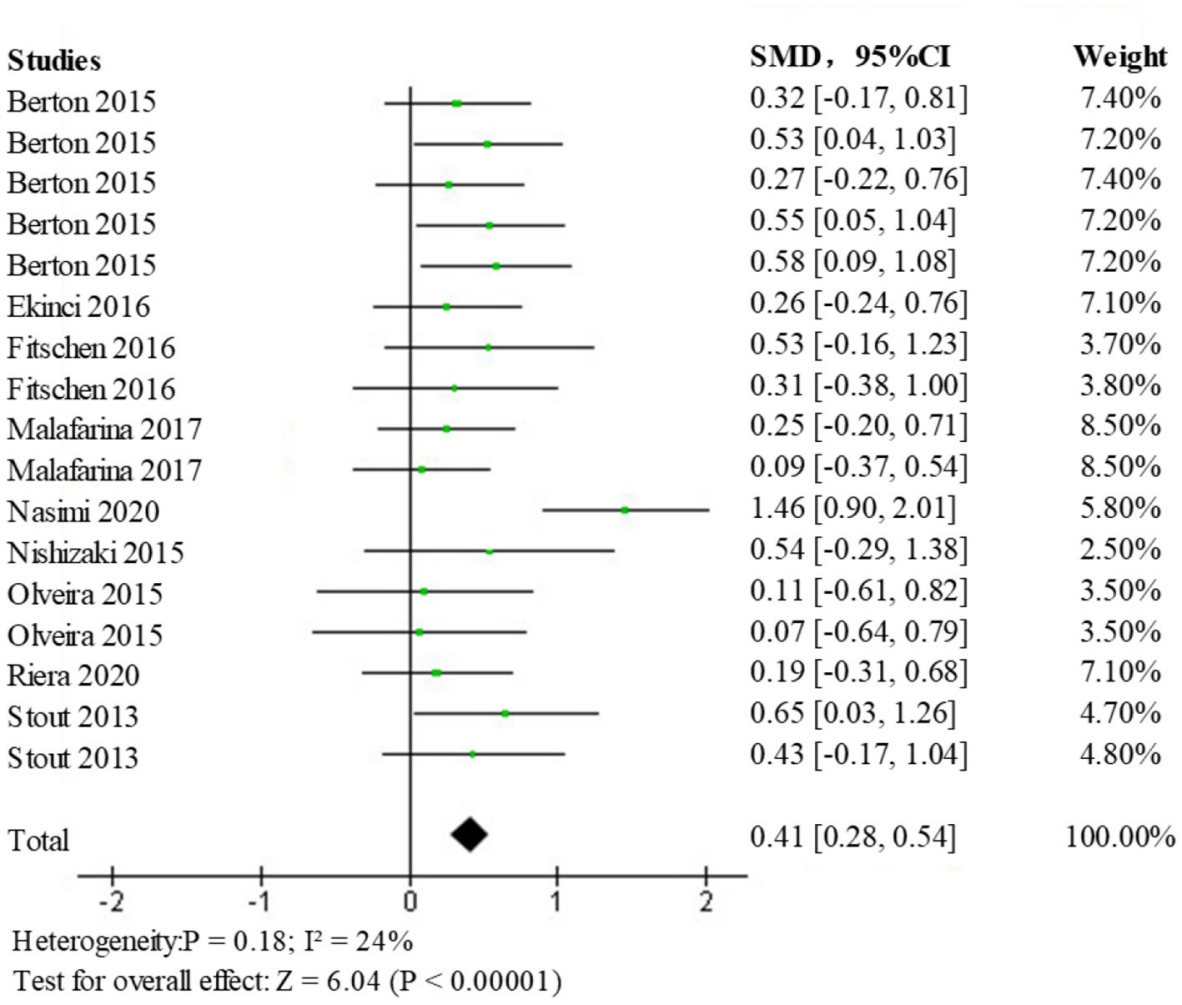
Forest diagram of the effect of HMB on muscle strength.

The fixed-effects model for analysis (SMD = 0.41; 95% CI: 0.28, 0.54; *p* < 0.00001) showed a statistically significant difference (*p* < 0.05) among the intervention vs. a control group of the elderly population with sarcopenia, the forest plot is shown in Figure 8. This indicates that the intervention group had a more positive effect on overall muscle strength than the control group, suggesting that HMB improves the overall muscle strength of patients with sarcopenia.

## Discussion

This systematic review and meta-analysis aimed to investigate the effects of HMB and HMB-containing supplements on the muscle strength of older people. Firstly, since the measurement of upper and lower extremity muscle strengths have different significance for physical mobility, we performed a comparative analysis of upper and lower extremity muscle strength, Both HMB and HMB-containing supplements were observed to have an enhancing effect on the upper and lower extremity muscle strength, but an improvement in the upper extremity muscle strength was better than the lower extremity muscle strength. In a meta-analysis by Courel et al. (10), a pooled analysis of 12 indicators that could reflect lower extremity muscle strength was carried out. It was concluded that HMB improved lower extremity muscle strength but had no significant effect.

The analysis of overall muscle strength showed that HMB and HMB-containing nutritional supplements enhance muscle strength in older adults. Subgroup analysis showed that subjects who received HMB and HMB-containing supplements had significantly greater muscle strength than those in the control subgroup. This finding has important clinical implications, as HMB or HMB-containing supplements alone have demonstrated a favorable safety profile with no significant increase in the incidence of adverse events compared to the placebo or conventional treatment groups. The current analysis of upper and lower extremity muscle strength lies in line with the previous findings, which further supports the use of HMB and HMB-containing supplements in the elderly population.

Multiple studies have explored the effects of HMB on muscle in different populations. A meta-analysis conducted by Rowlands et al. (11) evaluating the effects of HMB supplementation on muscle strength, body composition, and muscle damage in young men showed no effect of HMB supplementation on body composition. This is in contrast to our finding showing a positive effect of HMB on muscle mass in older adults. These differences in the findings suggest that different populations respond differently to HMB and HMB-containing supplements. This may be related to the androgen levels in men. A study has shown a relationship between muscle mass and various indicators of androgen status (12). Studies have shown that supplementing HMB can change the hormone concentration in the resting state, such as increasing the levels of growth hormone and insulin-like growth factor after training. At the same time, HMB can significantly increase the concentration of adrenocortico-tropic-hormone to reduce the catabolism of hormones. For the elderly with less exercise, HMB can effectively increase the male hormone content in the elderly, and the catabolism of hormones is significantly reduced, which significantly improves the muscle content and strength level (13). However, studies have shown that HMB intake does not increase the significantly increased hormone levels in the body for young people (14).

A meta-analysis of HMB supplementation with physical activity suggests that HMB combined with exercise improves muscle mass in older adults (10). In addition, results from a preliminary non-randomized controlled trial by Yamamoto et al. (15) in patients with sarcopenic gastric cancer showed that preoperative exercise routines (grip training, walking, and resistance training) at home coupled with nutritional support, including HMB were effective in alleviating sarcopenia and reducing postoperative complications. These are consistent with our findings. In conclusion, the findings of the present study show that supplementing HMB with appropriate exercise is effective in improving muscle strength and optimizing physical rehabilitation therapy.

## Data Availability Statement

The original contributions presented in the study are included in the article/supplementary material, further inquiries can be directed to the corresponding author.

## Author Contributions

All authors listed have made a substantial, direct, and intellectual contribution to the work and approved it for publication.

## Conflict of Interest

The authors declare that the research was conducted in the absence of any commercial or financial relationships that could be construed as a potential conflict of interest.

## Publisher's Note

All claims expressed in this article are solely those of the authors and do not necessarily represent those of their affiliated organizations, or those of the publisher, the editors and the reviewers. Any product that may be evaluated in this article, or claim that may be made by its manufacturer, is not guaranteed or endorsed by the publisher.
